# JUNGBRUNNEN1 Confers Drought Tolerance Downstream of the HD-Zip I Transcription Factor AtHB13

**DOI:** 10.3389/fpls.2017.02118

**Published:** 2017-12-15

**Authors:** Saghar Ebrahimian-Motlagh, Pamela A. Ribone, Venkatesh P. Thirumalaikumar, Annapurna D. Allu, Raquel L. Chan, Bernd Mueller-Roeber, Salma Balazadeh

**Affiliations:** ^1^Institute of Biochemistry and Biology, University of Potsdam, Potsdam, Germany; ^2^Max Planck Institute of Molecular Plant Physiology, Potsdam, Germany; ^3^Instituto de Agrobiotecnología del Litoral, CONICET-Universidad Nacional del Litoral, Facultad de Bioquímica y Ciencias Biológicas, Santa Fe, Argentina

**Keywords:** *Arabidopsis*, transcription factor, drought, JUB1, HB13

## Abstract

Low water availability is the major environmental factor limiting growth and productivity of plants and crops and is therefore considered of high importance for agriculture affected by climate change. Identifying regulatory components controlling the response and tolerance to drought stress is thus of major importance. The NAC transcription factor (TF) JUNGBRUNNEN1 (JUB1) from *Arabidopsis thaliana* extends leaf longevity under non-stress growth conditions, lowers cellular hydrogen peroxide (H_2_O_2_) level, and enhances tolerance against heat stress and salinity. Here, we additionally find that JUB1 strongly increases tolerance to drought stress in *Arabidopsis* when expressed from both, a constitutive (CaMV *35S*) and an abiotic stress-induced (*RD29A*) promoter. Employing a yeast one-hybrid screen we identified HD-Zip class I TF AtHB13 as an upstream regulator of *JUB1*. AtHB13 has previously been reported to act as a positive regulator of drought tolerance. AtHB13 and JUB1 thereby establish a joint drought stress control module.

## Introduction

Low water availability is the major environmental factor limiting growth and productivity in plants and crops. Global changes of climate will probably reduce the availability of water even more in a larger part of the world (Hamdy et al., 2003) increasing the need for drought tolerant crops. About 80–95% of the plant’s biomass is water and, thus, water is vital for plant growth and development. Soil water is taken up by the roots and transported through the xylem to leaves for various physiological processes including photosynthesis (Fang and Xiong, 2015). Eventually, water returns back to the atmosphere via transpiration.

Homeodomain-leucine zipper (HD-Zip) proteins are plant-specific transcription factors (TFs) encoded by 47 genes in *Arabidopsis thaliana*; they fall into four distinct groups defined by their primary sequences (HD-Zip I–IV). HD-Zip proteins have two functional domains: a homeodomain (HD) for DNA binding and a leucine zipper (Zip) domain located C-terminal to the HD and involved in protein–protein interactions (for homo- or heterodimerization). HD-Zip proteins participate in a variety of developmental processes and are involved in the plant’s response to environmental factors (Ariel et al., 2007; Harris et al., 2011).

In *Arabidopsis*, the HD-Zip class I family includes seventeen members with proteins harboring a well conserved HD domain and a less conserved Zip motif. HD-Zip I TFs typically bind to the dyad-symmetric sequence CAAT(A/T)ATTG (Palena et al., 1999; Johannesson et al., 2001; Capella et al., 2015), and they are mainly involved in the response to abiotic stresses, abscisic acid (ABA) and blue light treatment, and they affect seedling de-etiolation (Perotti et al., 2017).

Members of the HD-Zip I fall into six different clades, α to φ (Henriksson et al., 2005). γ-Clade HD-Zip I TFs are typically induced by ABA treatment and/or water deficit, and include *Arabidopsis AtHB7* and *AtHB12*, sunflower (*Helianthus annuus*) *HaHB4*, *Medicago truncatula MtHB1*, *Nicotiana attenuata NaHD20*, and rice *OsHOX6* (*Oryza sativa Homeobox 6*), *OsHOX22*, and *OsHOX24* (Harris et al., 2011). Experimental evidence shows that AtHB12 reduces growth during water deficit by inhibiting GA biosynthesis and thereby cell elongation (Son et al., 2010). Similarly, growth is reduced in *AtHB7* overexpressors, although no evidence for an involvement of GA was reported (Hjellström et al., 2003). Expression of the two paralogous genes is regulated in a coordinated manner, depending on the developmental stage of the plant and the environmental conditions (Ré et al., 2014).

Expression of the β-clade members *AtHB5* and *AtHB6* is also affected by water deficit and both genes appear to regulate growth in response to ABA treatment and/or water limitation (Söderman et al., 1999; Himmelbach et al., 2002; Henriksson et al., 2005). Furthermore, δ-clade genes *AtHB21*, *AtHB40*, and *AtHB53* are induced by ABA treatment and salinity stress; the three TFs are involved in controlling axillary bud development (González-Grandío et al., 2017).

*Arabidopsis AtHB13*, an α-clade HD-Zip I TF, is upregulated by low temperature, drought, and salinity, similar to its sunflower homologue *HaHB1*. Overexpression of both genes confers tolerance to these stresses which involves the stabilization of the cell membrane (Cabello et al., 2012; Cabello and Chan, 2012). Plants overexpressing *AtHB13* or *HaHB1* achieve an improved yield under normal and mild stress conditions suggesting both TFs may be employed as tools for establishing crops with enhanced tolerance to multiple stresses and increased yield (Cabello and Chan, 2012; Silva et al., 2016). Recently, *AtHB13* and its paralog *AtHB23* were shown to negatively affect stem elongation (Ribone et al., 2015).

TFs of the plant-specific NAC (NAM/ATAF/CUC) family play diverse roles in development and stress responses (Shao et al., 2015; Kim et al., 2016) and have been suggested as tools to improve stress tolerance in crops (Tran et al., 2010; Wang et al., 2016). NAC TFs harbor a well conserved, 60-amino acid-long N-terminal DNA-binding domain (NAM domain) and a variable downstream segment through which they interact with other proteins, including other NACs (to form heterodimers) or other regulatory proteins (Olsen et al., 2005).

JUNGBRUNNEN1 (JUB1; ANAC042) is a multifunctional member of the NAC TF family in *A. thaliana* acting as a negative regulator of senescence and a positive regulator of the tolerance to heat and salinity stress. While *JUB1* overexpressor (*JUB1Ox*) plants are tolerant to both stresses, the *jub1-1* knockdown mutant exhibits hypersensitivity (Shahnejat-Bushehri et al., 2012; Wu et al., 2012). JUB1 directly regulates the expression of stress-responsive TFs such as *DREB2A* and it reduces the cellular levels of reactive oxygen species (ROS), which contributes to the enhanced stress tolerance (Shahnejat-Bushehri et al., 2012; Wu et al., 2012). We and others recently reported that overexpression of *JUB1* enhances drought tolerance in both, tomato (Thirumalaikumar et al., 2017) and banana (Tak et al., 2017).

Besides its direct impact on stress regulatory genes, JUB1 also affects growth by negatively and directly regulating genes encoding key enzymes of gibberellin (GA) and brassinosteroid (BR) biosynthesis, namely *GA3ox1* and *DWF4*, respectively, as demonstrated for *Arabidopsis* (Shahnejat-Bushehri et al., 2016). Furthermore, overexpression of JUB1 leads to the accumulation of DELLA proteins which are master repressors of growth, but enhance stress tolerance (Davière and Achard, 2016; Shahnejat-Bushehri et al., 2016). We furthermore demonstrated that JUB1 exerts a conserved control over GA and BR metabolism and signaling genes in tomato (Shahnejat-Bushehri et al., 2017).

Here, we report *JUB1* as a drought-induced gene whose overexpression enhances drought tolerance in *Arabidopsis*. To unravel the regulatory integration of *JUB1* during drought stress we performed a yeast one-hybrid screen using a promoter fragment conferring H_2_O_2_- and drought-responsiveness to *JUB1*, and identified AtHB13 as its upstream transcription regulator. We demonstrate that AtHB13 confers its role in protecting plants against drought stress in part by regulating the expression of *JUB1*.

## Materials and Methods

### General

Oligonucleotides (**Supplementary Table S1**) were obtained from Eurofins MWG Operon (Ebersberg, Germany). Tools provided by the National Center for Biotechnology Information^1^, the Arabidopsis Information Resource^2^, the Plant Transcription Factor Database^3^, and PLAZA 3.0^4^ were used for computational analyses.

### Growth Conditions

*Arabidopsis thaliana* (L.) Heynh. (Col-0) was used as the wild type; transgenic lines are based on this accession. For experiments at seedling stage, seeds were surface sterilized and sown on half-strength Murashige and Skoog (MS) agar medium containing 1% (w/v) sucrose. Plants were grown in soil (Einheitserde GS90; Gebrüder Patzer) under a 12-h day (120 mmol m^-2^ s^-1^; 22°C) : 12-h night regime (22°C). For experiments shown in **Figures 4**, **5** and **Supplementary Figures S1**, **S2** plants were grown at 21°C under a 16-h day and 8-h dark regime. *35S:JUB1* (*JUB1Ox*), *RD29A:JUB1*, and *jub1-1* (SALK_ID 036474) plants were described previously (Wu et al., 2012). *AtHB13Ox* plants and *athb13* mutants were reported in (Cabello and Chan, 2012; Cabello et al., 2012).

### Constructs

Gene constructs were generated by polymerase chain reaction (PCR) and directional cloning. All PCR-generated amplicons were checked for correctness by DNA sequence analysis (Seqlab or LGC Genomics). Constructs were transformed into *Arabidopsis* via *Agrobacterium tumefaciens*-mediated transformation (floral dip method). To generate the *35S:AtHB13-GFP* and *AtHB13-GST* constructs, the *AtHB13* open reading frame (ORF) was PCR-amplified without the stop codon and cloned into pENTR/D-TOPO vector using the pENTR directional TOPO cloning kit (Invitrogen). The *AtHB13* ORF was then transferred to the GATEWAY vectors pK7FWG2 (Ghent University) and pDEST24 (Invitrogen), respectively, through LR recombination. To generate the *JUB1* promoter deletion constructs (*Pro*_*JUB*1_:GUS), 1.0, 0.73, 0.68, 0.31, and 0.21 kb long segments of the *JUB1* promoter were PCR-amplified and cloned into pENTR/D-TOPO vector using the pENTR directional TOPO cloning kit (Invitrogen). The promoter fragments were then transferred to the pKGWFS7 GATEWAY vector (Ghent University) by LR recombination. *pTUY1H-JUB1-373*: The selected 373-bp *JUB1* promoter region was first cloned via TA cloning into pCR2.1 entry vector (Invitrogen), and then transferred to the pTUY1H yeast transformation vector (with *LEU2* as selection marker) (Castrillo et al., 2011) by restriction enzyme-mediated cloning.

### Histochemical GUS Staining

Two-week-old seedlings were carefully transferred from agar media plates to Erlenmeyer flasks containing liquid MS medium (1% [w/v] sucrose) in the absence or presence of 10 mM H_2_O_2_, and incubated overnight. For drought treatments, 4-week-old soil-grown plants were not watered for 6 days, and leaves were harvested. Histochemical GUS staining was performed overnight at 37°C in the dark. Chlorophyll was removed by clearing the samples with 70% (v/v) ethanol. Quantification of GUS signal was done as described (Béziat et al., 2017).

### Quantitative Real-Time PCR (qRT-PCR)

Total RNA was extracted using the RNeasy Plant Mini kit (Qiagen, Hilden, Germany). Synthesis of cDNA and quantitative real-time PCR (qRT-PCR) using SYBR Green were performed as reported (Balazadeh et al., 2008, 2010) with *ACTIN2* (*At3g18780*) as the reference gene. Primer sequences are given in **Supplementary Table S1**. Primers were designed using the QuantPrime tool^5^ (Arvidsson et al., 2008).

### Yeast One-Hybrid Screen

The bait construct *pTUY1H-JUB1-373* (*LEU2* selection marker; *JUB1* promoter fragment upstream of *HIS3* reporter) was transformed into yeast strain Y187, mating type α. The mating-based Y1H screen was done using a library of approximately 1,200 *Arabidopsis* TFs, established in vector pDEST22 (*TRP1* selection marker) in yeast strain YM4271 (mating type a) (Castrillo et al., 2011). Screening for interaction between TFs and the 373 bp long *JUB1* promoter fragment was done on SD medium lacking the essential amino acids Leu, Trp, and His in the absence or presence of different concentrations of 3-amino-1,2,4-triazol (3AT) to prevent false positive interactions.

### Electrophoretic Mobility Shift Assay (EMSA)

AtHB13-GST fusion protein was purified from *Escherichia coli* expression strain BL21 Star (DE3) pRARE, which was generated by transforming the pRARE plasmid isolated from Rosetta (DE3) pRARE cells (Merck) into *E. coli* BL21 Star (DE3) (Invitrogen). Recombinant GST-fusion protein was purified using GST-agarose beads following the manufacturer’s instructions (Sigma–Aldrich, Taufkirchen, Germany). Electrophoretic mobility shift assays (EMSA) was performed as described (Wu et al., 2012) using the Odyssey Infrared EMSA kit (LI-COR). Sequences of 5′-DY682-labeled fragments are given in **Supplementary Table S1**.

### Chromatin Immunoprecipitation

Chromatin immunoprecipitation (ChIP) was carried out on chromatin extracted from *35S:AtHB13-GFP* plants grown (i) well-watered for 4 weeks, (ii) well-watered for 4 weeks and then drought stressed for 6 days (by withholding water), and (iii) well-watered for 50 days. WT plants grown in parallel served as controls in each experiment. Three independent ChIP experiments were performed. The qPCR primers for the *JUB1* promoter were designed to flank the AtHB13 binding site (**Supplementary Table S1**). As negative controls, primers annealing to promoter regions of two *Arabidopsis* genes lacking an AtHB13 binding site, i.e., *AT3G18040* (Neg 1) and *AT2G22180* (Neg 2), were used. ChIP-qPCR data were analyzed as described (Kaufmann et al., 2010).

### Determination of Ion Leakage

For ion leakage measurements, the first six leaves of the rosette were immersed in 10 ml deionized water and shaken at room temperature for 30 min. Electrical conductivity (σ1) was measured at 25°C, using a conductometer (Schott, Mainz, Germany). Then samples were boiled for 15 min, cooled down to 25°C, and conductivity (σ2) was measured again. Ion leakage was calculated through the expression σ1/σ2 × 100. Three independent experiments were performed.

### Determination of Relative Water Content (RWC)

Plant material (five leaves per genotype) was weighed (fresh weight, FW), then put in a Petri dish containing water and kept at room temperature for 3 h. Then, leaves were weighed again (turgid weight, TW). Relative water content (RWC) was calculated using the following formula: RWC [%] = (TW–FW)/(TW) × 100. Three independent experiments were performed.

### Establishment of Mild Drought Stress

Plants were grown at a 16-h light : 8-h dark cycle and well watered for 25 days. Thereafter, field capacity was maintained at 50% in all pots by adding the needed quantity of water. Field capacity was determined by weighting the pots. Before the experiment was started, pots were saturated and weighed (100% field capacity). During the experiment, pots were weighed each day and water was added in order to maintain the 50% of the field capacity. The amount of water added to each plant is shown in the figure panels.

### Determination of Relative Water Loss during Severe Drought Stress

Plants were grown at a 16-h light : 8-h dark cycle and drought experiments were started by stopping irrigation at day 20. After stopping irrigation, leaves were detached at the indicated times (see figures), weighed (W1), submerged in tap water for 3 h and weighed again (W2). Water loss [%] was calculated using the following formula: (W2–W1)/W2 × 100.

### AGI Codes

*ACTIN2* (*AT3G18780*), *JUB1* (*AT2G43000*), *ATHB13* (*AT1G69780*).

## Results

### Expression of *JUB1* Is Induced by Drought

The expression of *JUB1* rapidly increases after treatment of *Arabidopsis* plants with hydrogen peroxide (H_2_O_2_) or in the presence of different abiotic stresses such as salinity and heat (Wu et al., 2012; Allu et al., 2014; Shahnejat-Bushehri et al., 2016). To test whether *JUB1* is also induced by drought, we analyzed its expression in plants subjected to water shortage. To this end, 4-week-old wild-type Col-0 (WT) plants were subjected to water withholding for 6 days, and whole rosettes were harvested to quantify *JUB1* expression by qRT-PCR. As shown in **Supplementary Figure S1A**, *JUB1* expression was considerably higher in drought-stressed plants than in well-watered controls. We also established transgenic *Arabidopsis* plants expressing the *GUS* reporter gene from the 1-kb *JUB1* promoter (*Pro*_*JUB*1_:GUS plants). We grew *Pro*_*JUB*1_:GUS plants for 4 weeks under well-watered condition and then subjected them to drought stress (by stopping irrigation) for 6, 9, and 12 days. Histochemical GUS staining revealed drought-induced *JUB1* promoter activity (**Supplementary Figure S1B**).

### *JUB1* Confers Tolerance to Drought Stress in *A. thaliana*

**FIGURE 1:**
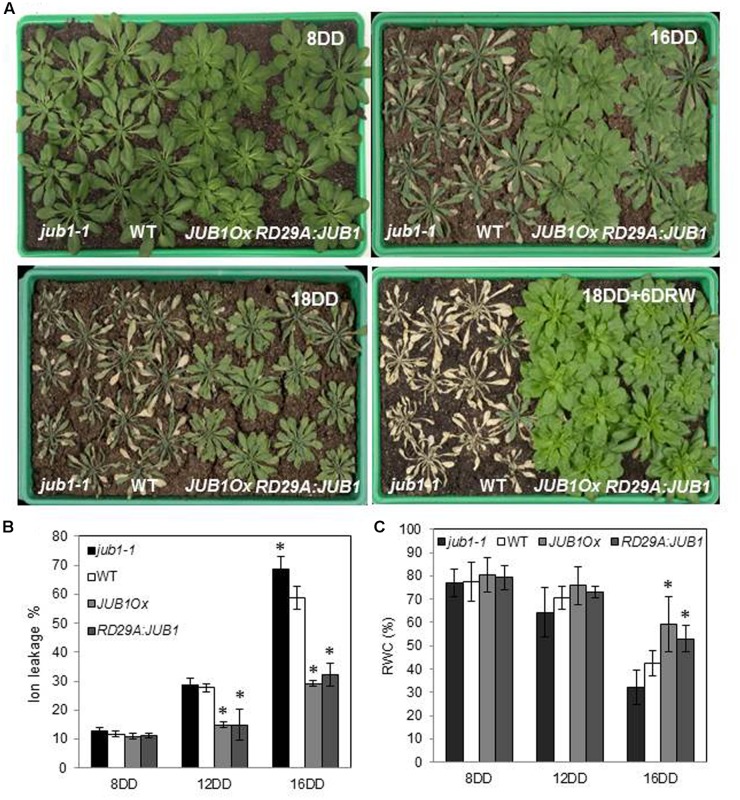
*JUB1* confers tolerance to drought. **(A)** Four-week-old *jub1-1*, WT, *JUB1Ox*, and *RD29A:JUB1* plants were grown in soil and subjected to drought stress by withholding water for 18 days. Photographs were taken 8 days after start of the drought stress experiment (8DD), 16DD, 18DD, and 18DD + 6 days of re-watering (18DD+6DRW). The experiment was repeated more than three times, and a representative result is shown here. **(B)** Ion leakage of the first six leaves of WT and transgenic lines after 8, 12, and 16 days of drought stress. **(C)** Relative water content (RWC) of leaves (%). Means ± SD are shown (*n* = 3). Asterisks represent statistically significant difference from WT; Student’s *t*-test (^∗^*p* < 0.05).

To investigate the function of JUB1 for the response to drought in *Arabidopsis*, we analyzed the phenotype of *JUB1* transgenic lines during drought stress. We previously reported that constitutive overexpression of *JUB1* results in reduced growth, delayed flowering, and late leaf senescence (Wu et al., 2012; Shahnejat-Bushehri et al., 2016); we here therefore included transgenic lines expressing *JUB1* under the control of the stress-inducible promoter *RESPONSIVE TO DESICCATION 29A* (*RD29A*) in our drought assays. The *RD29A* gene is highly responsive to drought, low temperature, high salt concentration, and desiccation (Yamaguchi-Shinozaki and Shinozaki, 1993, 1994) and only basal expression is observed in non-stressed plants (Yamaguchi-Shinozaki et al., 1999). However, as we reported previously, different *RD29A:JUB1* lines displayed different *JUB1* expression levels under control conditions (Wu et al., 2012). Here, we selected a *RD29A:JUB1* line with lowest expression of *JUB1* under non-stress condition for our analyses. The selected transgenic line displayed growth phenotypes similar to WT, in contrast to the plants strongly expressing *JUB1* from the constitutive CaMV *35S* promoter (*JUB1Ox*; Shahnejat-Bushehri et al., 2016). Four-week-old *RD29A:JUB1, JUB1Ox* and WT plants, as well as *jub1-1* knockdown mutants were dehydrated for 18 days. As shown in **Figure 1A**, both, *RD29A:JUB1* and *JUB1Ox* plants exhibited strong drought tolerance, while *jub1-1* and WT plants showed severe wilting after 16 days of drought (16DD), and were strongly dehydrated after 18 days (18DD). None of the WT and *jub1-1* plants recovered after 18 days of drought followed by re-watering for 6 days (18DD+6DRW) further demonstrating their sensitivity to drought stress. In contrast, *JUB1Ox* and *RD29A:JUB1* plants recovered rapidly after the 18 days of drought stress and showed complete survival after re-watering. Membrane stability under water deficit conditions was assessed by measuring ion leakage in both, control and treated plants. When water was withheld for 12 days, *jub1-1* and WT plants showed a higher ion leakage (∼30%) than *JUB1Ox* and *RD29A:JUB1* plants (∼15%). After 16 days of water withholding the differences in electrolyte leakage increased further; while *jub1-1* and WT plants showed an ion leakage of ∼70 and ∼58%, respectively, ion leakage was only ∼30% for both, *RD29A:JUB1* and *JUB1Ox* plants upon stress (**Figure 1B**).

Furthermore, RWC in leaves was determined after 8, 12, and 16 days of withholding water (**Figure 1C**). RWC was not significantly different between the genotypes at the early stage of drought (8DD), while at later stages of drought (16DD) a significantly higher RWC was observed in *RD29A:JUB1* and *JUB1Ox* plants than in WT and *jub1-1* plants (**Figure 1C**). Collectively, our results reveal that *JUB1*, when expressed from the constitutive CaMV *35S* promoter or from the abiotic stress-induced *RD29A* promoter, confers superior tolerance to drought stress in *Arabidopsis*.

### A Promoter Region Central for Drought-Induced Expression of *JUB1*

**FIGURE 2:**
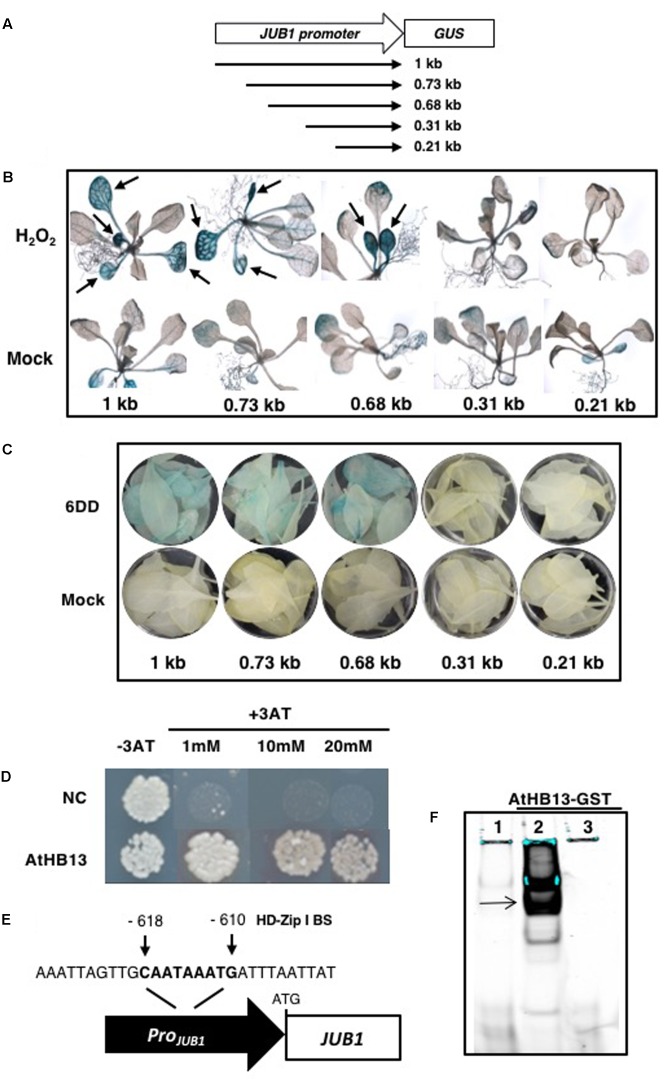
Identification of an upstream regulator of *JUB1*. **(A)** A series of 5′ deletions of the *JUB1* promoter (including the 5′- untranslated region up to the ATG start codon) were transcriptionally fused to the β-*GLUCURONIDASE* (*GUS*) reporter gene and the constructs were transformed into *Arabidopsis*. **(B)** Two-week-old seedlings of different *Pro*_*JUB*1_:GUS deletion lines (1 kb as well as 0.73, 0.68, 0.31, and 0.21 kb) were subjected to H_2_O_2_ (10 mM) treatment overnight and then incubated at 37°C in GUS buffer. Arrows indicate induced GUS staining. Note the lack of induction of GUS activity in the –0.31-kb and –0.21-kb deletion lines. **(C)** Four-week-old *Pro*_*JUB*1_:GUS deletion lines were subjected to drought for 6 days (6DD). Following GUS staining, the lines expressing *GUS* from the 1, 0.73, and 0.68 kb *JUB1* promoter fragments showed higher GUS activity than the corresponding well-watered (control) plants, while no GUS staining was visible in the –0.31-kb and –0.21-kb deletion lines. **(D)** Yeast-one-hybrid (Y1H) assay demonstrates interaction between the functional 373-bp *JUB1* promoter fragment and transcription factor (TF) AtHB13. The *JUB1* promoter fragment contains the common binding site of HD-Zip I TFs at positions –618 to –610 bp upstream of the translational start site (ATG). Upon interaction of AtHB13-GAL4AD fusion protein with the binding site, transcription of the yeast *HIS3* reporter gene is activated and diploid yeast cells grow on SD medium lacking the three essential amino acids Trp, Leu, and His. The yeast one-hybrid assay was performed three times giving the same result. NC, negative control containing the *pTUY1H-JUB1-373* plasmid but no TF as a test for autoactivation. **(E)** Schematic representation of the HD-Zip I binding site (BS) within the *JUB1* promoter. The sequence of the BS as well as the surrounding nucleotides are indicated. **(F)** EMSA showing binding of purified AtHB13-GST protein to the *JUB1* promoter region harboring the HD-Zip I BS. DNA binding reactions were performed with a 40-bp long wild-type fragment derived from the *JUB1* promoter containing the HD-Zip I BS. 1, 5′-DY682-labeled, double-stranded oligonucleotide; 2, labeled probe plus AtHB13-GST protein; 3, labeled probe plus AtHB13-GST and 200× competitor (unlabeled oligonucleotide).

We intended to identify upstream regulatory factors controlling the expression of *JUB1* during abiotic stress. To this end, we performed a deletion analysis of the *JUB1* promoter and then conducted a yeast one-hybrid (Y1H) screen to identify TFs binding to a functionally relevant promoter segment. Various 5′ deletions of the *JUB1* promoter were transcriptionally fused to the *Escherichia coli β-GLUCURONIDASE* (*GUS*) reporter gene and the constructs were transformed into *Arabidopsis* (**Figure 2A**). The promoter-reporter lines (hereafter, *Pro*_*JUB*1_:GUS deletions) were subjected to H_2_O_2_ and drought treatments to identify a promoter region relevant for the response of *JUB1* to these stresses. To this end, (i) 2-week-old seedlings of *Pro*_*JUB*1_:GUS deletion lines were treated with 10 mM H_2_O_2_ and (ii) 4-week-old *Pro*_*JUB*1_:GUS plants were subjected to desiccation for 6 days (6DD) and leaves were harvested for analysis. Histochemical GUS staining revealed that the 1-kb *JUB1* promoter as well as the 0.73-kb and 0.68-kb deletion variants, but not the 0.31-kb and the 0.21-kb promoters, confer stress-inducible activation of the *GUS* reporter gene, indicating that the region responsive to H_2_O_2_ and drought is located between positions -0.68 and -0.31 kb of the *JUB1* promoter (**Figures 2B,C** and **Supplementary Figure S1C**).

### AtHB13 Binds to the *JUB1* Promoter and Activates Its Expression during Drought Stress

To identify upstream transcriptional regulators of *JUB1*, we performed a yeast one-hybrid (Y1H) screen using the 0.37-kb promoter fragment (-0.68 and -0.31 kb upstream of the *JUB1* start codon) involved in the H_2_O_2_- and drought responsiveness of the *JUB1* gene as bait. In a screen with nearly 1,200 *Arabidopsis* TFs we identified HD-Zip I protein AtHB13 (AT1G69780) as a TF binding to the abiotic stress-responsive segment of the *JUB1* promoter (**Figure 2D**). As AtHB13 has previously been reported to affect the response of *A. thaliana* to various biotic and abiotic stresses including drought (Cabello and Chan, 2012; Cabello et al., 2012; Gao et al., 2014), we investigated whether it is a genuine upstream regulator of *JUB1*.

**FIGURE 3:**
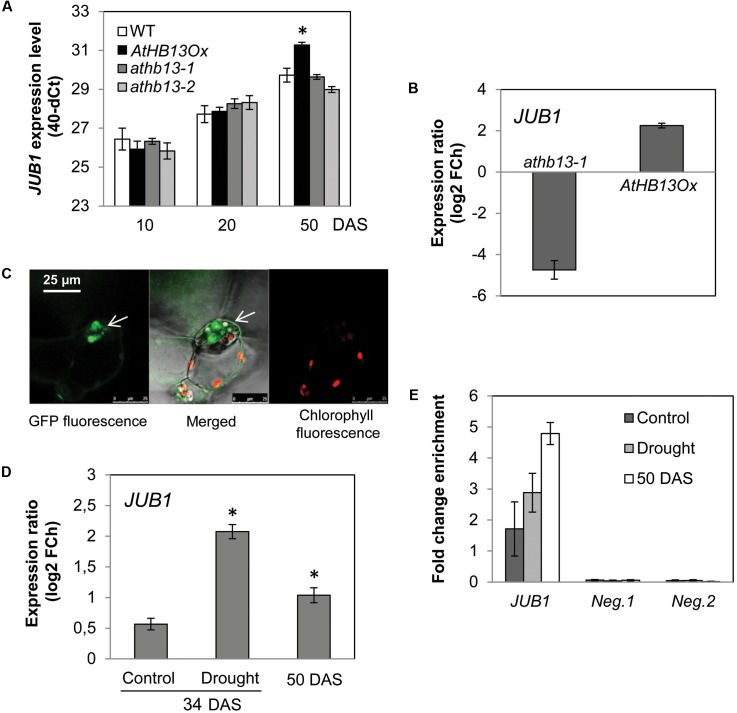
AtHB13 directly regulates *JUB1*. **(A)** Expression of *JUB1* in 10-, 20-, and 50-day-old WT, *AtHB13Ox*, and *athb13-1* and *athb13-2* plants in well-watered condition. Transcript levels were determined by qRT-PCR; values are expressed as the difference between an arbitrary value of 40 and dCt, so that high 40-dCt value indicates high gene expression level. Means ± SD calculated from three independent biological experiments (each with nine leaves pooled from three plants). Expression levels were normalized against the expression level of *ACTIN2*. DAS, days after sowing. Asterisk indicates statistically significant difference (Student’s *t*-test (^∗^*p* < 0.05) from WT. **(B)** Expression of *JUB1* in *35S:AtHB13* (*AtHB13Ox*) and *athb13-1* plants compared to WT upon drought treatment. For drought treatment, 4-week-old plants were subjected to water withholding for 6 days. Whole rosettes of drought-treated and well-watered (control) plants were harvested for gene expression analysis by qRT-PCR. Data represent the means of three biological repetitions ± SD. FCh, fold change. **(C)** Confocal microscope image showing nuclear localization of the AtHB13-GFP fusion protein in transgenic *35S:AtHB13-GFP Arabidopsis* plants. Left, GFP signal; middle, chlorophyll autofluorescence merged with GFP fluorescence; right, chlorophyll autofluorescence. **(D)** Expression of *JUB1* in *35S:AtHB13-GFP* plants compared to WT upon drought stress and at a later stage of development (50-day-old plants). For drought treatment, 4-week-old plants were subjected to drought by withholding water for 6 days. Whole rosettes of drought-treated and well-watered (control) plants were harvested for gene expression analysis by qRT-PCR. Data represent the means of three biological repetitions ± SD. FCh, fold change. Asterisks indicate statistically significant difference (*p* < 0.01; Student’s *t*-test) from the non-stress control at 34 DAS. **(E)** ChIP-qPCR showing enrichment of the *JUB1* promoter region containing the HD-Zip I binding site, quantified by qPCR. For the ChIP experiment rosettes of *35S:AtHB13-GFP* and WT plants were harvested as follows: from 4-week-old control plants (well watered; ‘control’); from plants grown for 4 weeks in well-watered condition and then subjected for 6 d to drought stress by withholding water (‘drought’); and from 50-day-old plants grown under well-watered condition (‘50 DAS’). As negative controls, primers annealing to promoter regions of two *Arabidopsis* genes lacking an HD-Zip I binding site, i.e., *AT3G18040* (*Neg. 1*) and *AT2G22180* (*Neg. 2*), were used. Data represent the means of three biological repetitions ± SD.

Interestingly, the 373-bp *JUB1* fragment employed in the Y1H assay harbors an HD-Zip class I binding site, namely CAATAAATG (**Figure 2E**). The motif is identical to the one reported by Sessa et al. (1993) for AtHB1 (another HD-Zip I protein from *Arabidopsis*), with the exception of the underlined A which is a T in the AtHB1 binding site. To test whether AtHB13 binds to the CAATAAATG sequence within the frame of the *JUB1* promoter, we performed EMSAs using recombinant AtHB13-GST (glutathione *S*-transferase) fusion protein and a 5′-DY682-labeled 40-bp *JUB1* promoter fragment harboring the HD-Zip class I binding site. As shown in **Figure 2F**, AtHB13 protein physically interacts *in vitro* with the respective *JUB1* promoter fragment. Next, we checked expression of *JUB1* in rosette leaves of transgenic plants overexpressing *AtHB13* from the CaMV *35S* promoter (*AtHB13Ox* lines; Cabello et al., 2012) and WT plants at different developmental stages, i.e., at 10, 20, and 50 days after sowing (DAS). Interestingly, expression of *JUB1* was not altered in the *AtHB13Ox* plants at 10 and 20 DAS, but was significantly upregulated in *AtHB13Ox* plants at 50 DAS (**Figure 3A**). Thereafter, we analyzed the expression of *JUB1* in *AtHB13Ox* overexpressors and *athb13* knockout mutants (Cabello and Chan, 2012; Cabello et al., 2012) during drought stress. To this end, 4-week-old plants were subjected to 6 days of water withholding. As shown in **Figure 3B**, expression of *JUB1* was enhanced in *AtHB13Ox* plants compared to WT, and strongly repressed in the *athb13-1* mutant upon drought stress. Collectively, our data indicate that AtHB13 functions as a positive regulator of *JUB1* expression at later stages of development and upon drought stress.

To confirm that AtHB13 interacts with the *JUB1* promoter *in planta*, we performed ChIP assays using *Arabidopsis* plants stably expressing GFP-tagged AtHB13 protein (hereafter, *35S:AtHB13-GFP*). Analysis by confocal microscopy revealed nuclear localization of AtHB13-GFP protein, in accordance with the biological function of AtHB13 as a transcriptional regulator (**Figure 3C**). Next, we harvested rosette leaves from *35S:AtHB13-GFP* and WT plants for gene expression analysis and ChIP-qPCR assays. Plants were grown (i) for 4 weeks in well-watered condition (control); (ii) for 4 weeks in well-watered condition, followed by 6 days without watering (drought); (iii) for 50 days in well-watered condition (50 DAS). Under control condition, *JUB1* expression was similar in *35S:AtHB13-GFP* and WT plants, while its expression was significantly induced under drought stress in *35S:AtHB13-GFP* compared to WT. *JUB1* expression was also elevated, although less strongly, in well-watered 50 DAS *35S:AtHB13-GFP* plants (**Figure 3D**). ChIP-qPCR revealed enrichment of the *JUB1* promoter fragment harboring the AtHB13 binding site, in particular at 50 DAS control and in drought stress conditions (**Figure 3E**).

### AtHB13 Confers Drought Tolerance in Part via Regulation of *JUB1*

Expression of *AtHB13* and of its sunflower homologue *HaHB1* is induced by water deficit and overexpression of the two TFs in transgenic *Arabidopsis* plants improves drought tolerance (Cabello and Chan, 2012), similar to the overexpression of *JUB1* reported here. Considering the fact that AtHB13 binds the *JUB1* promoter, and regulates *JUB1 in planta*, the higher drought tolerance of *AtHB13* overexpressors might be conveyed through an upregulation of *JUB1* expression by the HD-Zip I TF. To test this hypothesis, we tested the drought tolerance of *AtHB13Ox*, *JUB1Ox*, *athb13-1*, *athb13-2*, *jub1-1*, and WT plants grown side by side. Plants were grown under well-watered condition and drought stress was started at day 20, before bolting occurred in all genotypes. Thereafter, irrigation was stopped, which gradually produced severe drought stress. **Supplementary Figure S2A** shows the phenotype of all plants after eight days of drought stress indicating that the two TF overexpressors (*AtHB13Ox*, *JUB1Ox*) are more tolerant than the WT, while the *athb13-1*, *athb13-2*, and *jub1-1* mutants are less tolerant. We determined water loss in plants of all genotypes at different days after stopping irrigation (**Supplementary Figure S2B**). Our results show that water loss was significantly lower in *JUB1Ox* than WT plants, in accordance with the observed increase in drought tolerance of these lines, while *jub1-1* and *athb13* mutants performed slightly worse than the WT in the experiments performed (**Supplementary Figures S2B,C**). Similar conclusions can be drawn from water consumption experiments; as shown in **Supplementary Figures S2D,E**, overexpressors consumed less water than WT and mutant plants during mild drought stress, although the reduction in water consumption was much more prominent in *JUB1Ox* than *AtHB13Ox* plants, which may in part be due to the more compact growth phenotype of *JUB1* overexpressors compared to *AtHB13* overexpressors.

**FIGURE 4:**
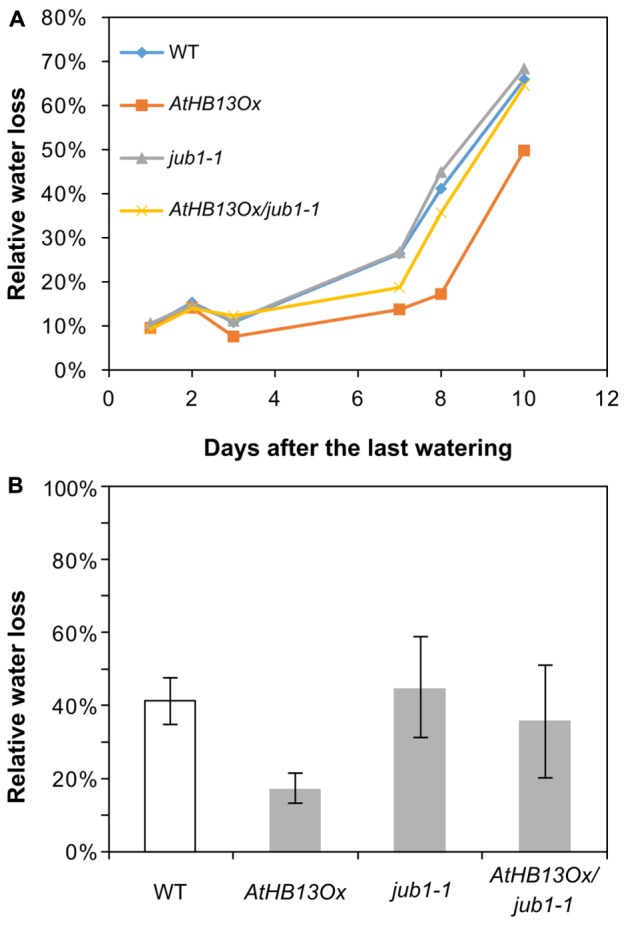
*AtHB13Ox*/*jub1-1* plants behave similar to *jub1-1* plants during drought stress. **(A)** Kinetics of relative water loss of leaves form WT, *AtHB13Ox*, *jub1-1*, and *AtHB13Ox*/*jub1-1* plants. Plants were grown under well-watered condition for 20 days; thereafter, irrigation was stopped, gradually leading to severe drought stress. **(B)** Relative water loss in leaves during 9 days of the treatment. For each genotype, leaves from five plants (one leaf per plant) were analyzed. Bars represent SD. The asterisk indicates significant difference from *AtHB13Ox*/*jub1-1* plants (Student’s *t*-test, *p* < 0.05).

To test whether AtHB13 requires *JUB1* for improved drought tolerance we crossed the *AtHB13Ox* plant with the *jub1-1* mutant. As seen in **Figure 4A**, water loss in the *AtHB13Ox*/*jub1-1* line was similar to that of the *jub1-1* mutant and the WT, while water loss was much lower in the *AtHB13Ox* plant, strongly indicating that AtHB13 confers drought tolerance at least in part through transcriptional control of *JUB1* (**Figure 4B**).

## Discussion

The NAC TF JUNGBRUNNEN1 (JUB1) has originally been identified as a positive regulator of leaf longevity in *A. thaliana*, and to enhance the tolerance toward heat and salinity stress when overexpressed (Wu et al., 2012; Shahnejat-Bushehri et al., 2012). Here, we show that overexpression of *JUB1* in transgenic *Arabidopsis* also enhances tolerance to drought stress. Of importance, this capacity of JUB1 is observed in both, transgenic plants expressing *JUB1* from the constitutive CaMV *35S* promoter and the stress-induced *RD29A* promoter. This is an important notion as constitutive overexpression of *JUB1* affects plant morphology, due to the fact that JUB1 negatively controls the expression of two key genes of phytohormone biosynthesis, namely GAs (by inhibiting *GA3ox1*) and BRs (by inhibiting *DWF4*). However, when *JUB1* is expressed from a stress-inducible promoter, no major developmental differences to wild-type plants are observed (Wu et al., 2012), while tolerance to salinity and heat stress is retained, indicating that the developmental effect of JUB1 can be largely separated from the stress tolerance effect. A similar observation we made here with respect to drought tolerance gained through JUB1: while plants expressing *JUB1* from the constitutive CaMV *35S* promoter are smaller and more compact than wild-type plants (Shahnejat-Bushehri et al., 2016; **Figure 1A** and **Supplementary Figure S2A**), development of *RD29A:JUB1* plants is virtually indifferent from that of the wild type (**Figure 1A**). However, strong drought tolerance is observed in both transgenic lines, irrespective of the growth phenotype (**Figure 1**). This observation indicates that JUB1 can improve tolerance to drought without limiting growth to a large extent.

We have previously reported that expression of *JUB1* is rapidly induced by H_2_O_2_ treatment (Wu et al., 2012). We further observed that overexpression of *JUB1* in transgenic *Arabidopsis* plants lowers the level of cellular H_2_O_2_, suggesting that this TF dampens H_2_O_2_ accumulation through a gene regulatory network that is currently not known in its details. However, JUB1 acts as a direct upstream transcriptional regulator of *DREB2A* (*DEHYDRATION-RESPONSIVE ELEMENT-BINDING PROTEIN 2A*), which encodes an AP2-type TF well known for its involvement in regulating heat and drought responses (Sakuma et al., 2006; Kant et al., 2008). DREB2A itself controls the expression of *Heat shock factor A2* (*HsfA2*) and through this affects several *HEAT SHOCK PROTEIN* (*HSP*) genes and genes for H_2_O_2_ scavenging enzymes (Schramm et al., 2008; Yoshida et al., 2008). The reduced H_2_O_2_ level of *JUB1* overexpressor plants may also be causative for their strongly enhanced drought tolerance. The fact that *JUB1* is induced by H_2_O_2_ in conjunction with the observation that JUB1 dampens cellular H_2_O_2_ level suggests the presence of a regulatory loop that helps to protect the plant against overshooting cellular H_2_O_2_ levels.

**FIGURE 5:**
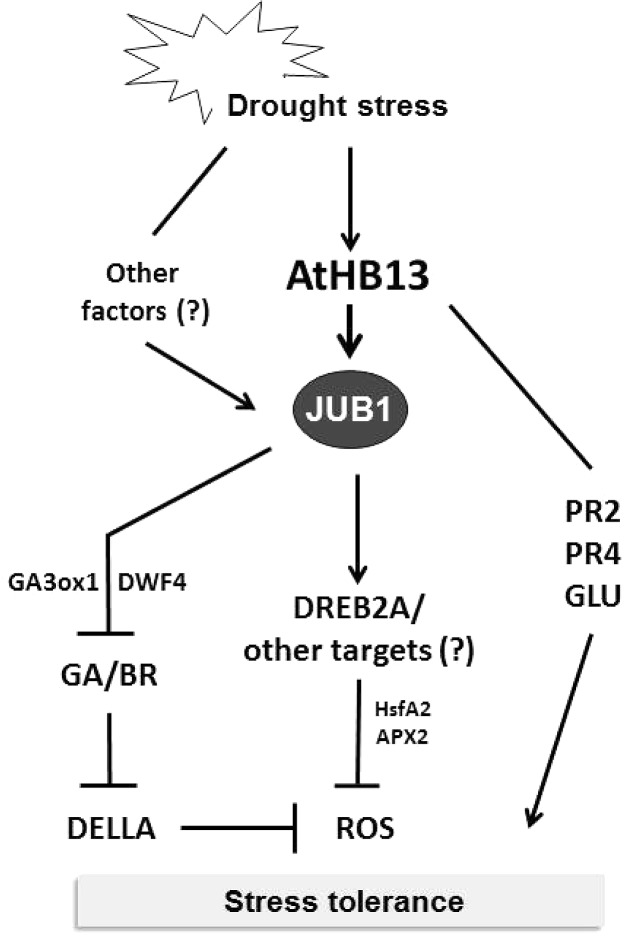
Model for AtHB13-*JUB1* regulation of drought stress tolerance. Drought stress induces the expression of both, *AtHB13* and *JUB1*. Increased levels of JUB1 confer enhanced tolerance to drought, in part by lowering cellular reactive oxygen species (hydrogen peroxide) level and by restricting growth via the GA/BR/DELLA pathway. AtHB13 also indirectly induces the expression of glucanase (*PR2* and *GLU*) and chitinase (*PR4*) genes, each of which enhances drought tolerance when overexpressed. However, control of the *PR* and *GLU* genes appears to be independent of JUB1.

To identify upstream transcriptional regulators controlling the H_2_O_2_-dependent activation of *JUB1*, we performed a yeast one-hybrid screen and identified AtHB13, an HD-Zip I TF, as a positive regulator of *JUB1* expression. Notably, the part of the *JUB1* promoter that controls responsiveness to H_2_O_2_ also controls the responsiveness to drought, suggesting that the drought responsiveness of *JUB1* is mediated through H_2_O_2_ which accumulates in drought-stressed plants (Kar, 2011; You and Chan, 2015). AtHB13 binds to the CAATAAATG element present within the relevant *JUB1* promoter segment. Of importance, AtHB13 has been reported previously to improve drought tolerance when overexpressed in transgenic *Arabidopsis* plants (Cabello and Chan, 2012). In addition, it was shown that AtHB13 induces the expression of *PATHOGENESIS RELATED2* (*PR2*) and *PR4*, genes that are able to individually confer drought tolerance (Cabello and Chan, 2012). However, in our experiments transcript levels of *PR2* and *PR4* were not induced by JUB1 (data not shown), indicating that the drought tolerance conferred by AtHB13 occurs through at least two different mechanisms, one of which involves JUB1 (**Figure 5**).

Recently, we demonstrated enhanced tolerance to drought stress in transgenic tomato (*Solanum lycopersicum*) plants overexpressing *JUB1* from *Arabidopsis*. In contrast, inhibition of tomato *JUB1* (*SlJUB1*) by virus-induced gene silencing significantly lowered drought tolerance associated with an increase in the level of H_2_O_2_, and a decrease of the expression of various drought-responsive genes including *SlDREB1, SlDREB2*, and *SlDELLA* (Thirumalaikumar et al., 2017). Similarly, banana (*Musa acuminata*) plants overexpressing *MusaNAC042* (the closest homologue of *JUB1* in this species) revealed increased tolerance to drought stress (Tak et al., 2017). Although levels of H_2_O_2_ were not determined in this study, the observation that *MusaNAC042* overexpressors contain lower levels of malondialdeyhde (MDA, a marker of lipid peroxidation) than wild-type plants indicates reduced stress-induced oxidative damage (Tak et al., 2017). The molecular control network through which MusaNAC042 lowers oxidative stress damage is unknown at present.

An interesting observation we made is the following: while overexpressing *AtHB13* triggers elevated expression of *JUB1* in older plants (50 DAS), it does not do so in younger plants (10 DAS; **Figure 3A**). Furthermore, while *JUB1* is not much affected by AtHB13 in well-watered plants, its expression increases in *AtHB13Ox* plants compared to WT when plants are subjected to drought stress (**Figure 3D**). Finally, while strong constitutive overexpression of *JUB1* reduces growth (Shahnejat-Bushehri et al., 2016), this is not the case for *AtHB13* overexpressors (Cabello et al., 2012; Cabello and Chan, 2012), although AtHB13 positively controls *JUB1* expression. This indicates that elevated levels of AtHB13 *per se* may not be sufficient to enhance *JUB1* expression under all conditions and suggests that additional mechanisms are needed to trigger transcriptional activation of *JUB1* by AtHB13. There are several principle mechanisms that could make AtHB13 competent for activating *JUB1*, including the following: (i) HD Zip TFs often form heterodimers with other family members in a selective manner (Johannesson et al., 2001; Capella et al., 2015) or with TFs or other families. It may thus be possible that AtHB13 interacts with other TFs at the later stages of leaf development or during drought stress. A possible candidate might be AREB3, a bZIP TF that interacts with AtHB13^6^ and is involved in ABA-dependent signaling and the response to drought stress (Uno et al., 2000). Importantly, *JUB1* expression is strongly reduced in the *athb13* knockout mutant, clearly indicating that AtHB13 is necessary but not potentially sufficient for full expression of *JUB1*. (ii) TFs of other families may be required in addition to AtHB13 for enhanced expression of *JUB1* under drought stress. (iii) The activity and/or stability of HD Zip TFs may be regulated by posttranslational modification such as phosphorylation, as for example reported for the HD Zip II TF HAT1 in *Arabidopsis* (Zhang et al., 2014). In a similar way, the stability of the AtHB13 protein may be enhanced in later stages of leaf development or in drought stress conditions. (iv) A TF suppressing *JUB1* expression might be active in young leaves, thereby overriding the activation by AtHB13 before the leaves enter a more mature stage. Which of these mechanisms are realized in *Arabidopsis* has to be revealed in further studies.

## Author Contributions

SB and BM-R conceived the idea for the study. SE-M performed the *JUB1* promoter deletion study and the Y1H screen. SE-M and VT performed the drought stress experiments. PR made the crosses, confirmed their genotypes, and performed drought stress experiments under the supervision of RC. VT generated the *35S:AtHB13-GFP* lines and performed confocal microscopy to check nuclear localization of AtHB13; he also prepared the *AtHB13-GST* construct. ChIP experiments were done by SE-M. AA and VT performed the AtHB13-GST protein purification and EMSA experiments. SB and BM-R wrote the manuscript, with contributions from SE-M and RC.

## Conflict of Interest Statement

The authors declare that the research was conducted in the absence of any commercial or financial relationships that could be construed as a potential conflict of interest.

## Footnotes

1 http://www.ncbi.nlm.nih.gov/

2 http://www.arabidopsis.org/

3 http://plntfdb.bio.uni-potsdam.de/v3.0/

4 http://bioinformatics.psb.ugent.be/plaza/

5 http://quantprime.mpimp-golm.mpg.de/

6 https://bar.utoronto.ca/eplant/

## Supplementary Material

**FIGURE S1 |** Histochemical GUS staining of *Pro*_*JUB*1_:GUS lines upon drought stress. **(A)** Expression level of *JUB1* in leaves of 4-week-old WT plants subjected to 6 days of dehydration (6DD) compared to well-watered (Mock) plants. Transcript levels were determined by qRT-PCR and normalized applying the dCt method. Values are expressed as the difference between an arbitrary value of 40 and dCt, so that high 40 – dCt value indicates high gene expression level. Means ± SD calculated from three independent biological replicates, with nine leaves from three plants in each replicate. *ACTIN2* was used as reference gene. Asterisks indicate significant difference in the level of *JUB1* between 6DD and mock-treated samples (*p* < 0.05; Student’s *t*-test). **(B)** Induction of *JUB1* promoter by drought stress, shown by histochemical GUS staining in leaves of *Arabidopsis Pro*_*JUB*1_:GUS plants (1000-bp *JUB1* promoter) plants. Plants were grown for 4 weeks under well-watered conditions and then irrigation was stopped for 6, 9, and 12 days. **(C)** Quantification of GUS signal in leaves of 14-day-old *Pro*_*JUB*1_:GUS seedlings drought-stressed on filter paper for 2 h (‘Drought’) or kept in humidified condition (‘Mock’). Promoters of 0.68 and 0.31 kb length were analyzed. GUS signal was quantified as reported (Béziat et al., 2017) by selecting rectangular areas of maximum size of individual leaves. High gray values represent elevated GUS staining. Means ± SD of 20 leaves from 6 to 8 seedlings each.

**FIGURE S2 |** Behavior of transgenic *JUB1* and *AtHB13* plants during water deficit. **(A)** Phenotype of *JUB1* and *AtHB13* transgenic lines under severe drought stress. WT, *JUB1Ox*, *jub1-1*, *AtHB13Ox*, *athb13-1*, and *athb13-2* plants grown in well-watered condition for 20 days and then subjected to severe drought stress for 8 days by completely stopping watering at day 20. **(B)** Kinetics of relative water loss of leaves from wild-type (WT) and transgenic *Arabidopsis* plants. **(C)** Relative water loss in leaves after day 8 of drought stress. For each genotype, leaves from five plants (one leaf per plant) were analyzed. Error bars represent SD. Plants in **(B,C)** were grown as in **(A)**. **(D)** Kinetics of water consumption in WT and transgenic (*jub1-1*, *JUB1Ox, AtHB13Ox*) plants during mild drought stress. Plants were grown in soil under well-watered condition until day 25. Then, field capacity was maintained at 50% in all pots by adding the needed quantity of water. The volume of water added to each plant is shown. The graph represents the average of three experiments ± SD calculated from data obtained from five plants per genotype in each experiment. **(E)** Total amount of water added to each plant line until the end of the experiment. The graph represents the average of three independent experiments ± SD calculated from data obtained from five plants per genotype in each experiment. Asterisks in **(C,E)** indicate significant difference from WT (Student’s *t*-test; *p* < 0.05).

**TABLE S1 |** Constructs and primer sequences.
