# Supplementary figures and images for: JUNGBRUNNEN1 Confers Drought Tolerance Downstream of the HD-Zip I Transcription Factor AtHB13
Source: Front Plant Sci. 2017 Dec 15;8:2118. doi: 10.3389/fpls.2017.02118 (PMC5736527; doi:10.3389/fpls.2017.02118)

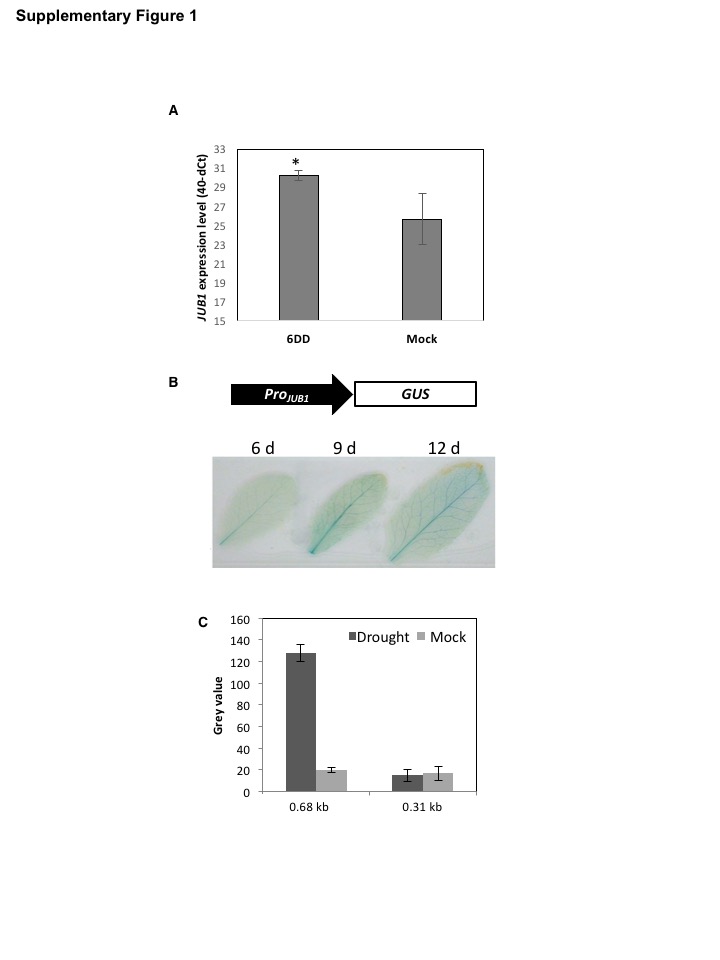

Supplement: Supplementary file 1 [file Image_1.JPEG]

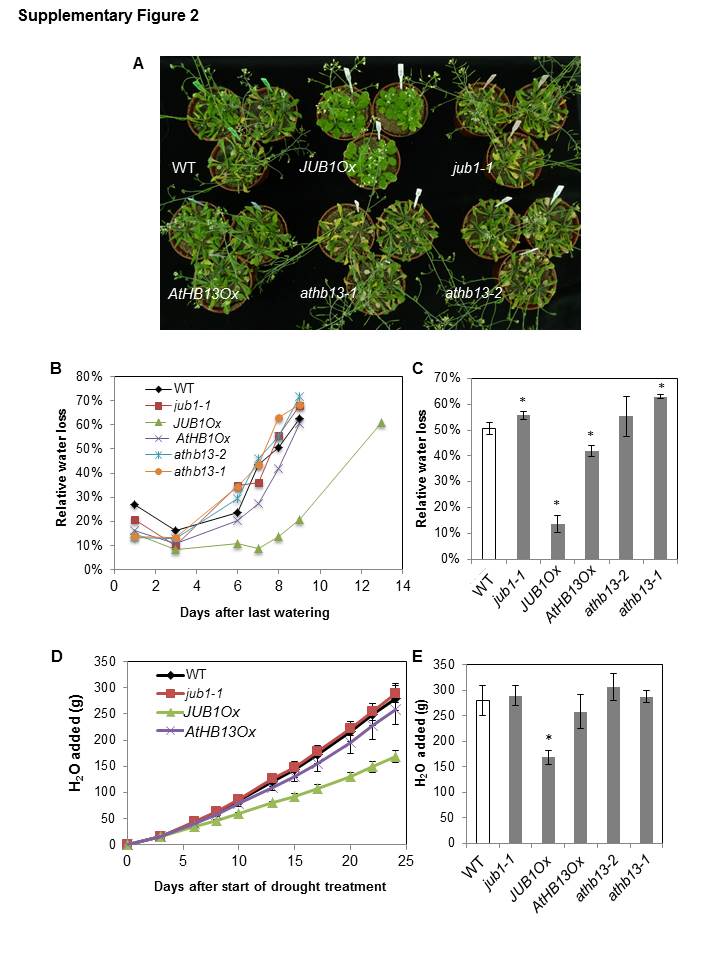

Supplement: Supplementary file 2 [file Image_2.JPEG]
